# Associations of diabetes mellitus and hypertension with adherence to continuous positive airway pressure therapy in male patients with obstructive sleep apnea

**DOI:** 10.20407/fmj.2020-028

**Published:** 2021-08-20

**Authors:** Shigeko Kojima, Ayako Saito, Fumihiko Sasaki, Masamichi Hayashi, Yuki Mieno, Hiroki Sakakibara, Shuji Hashimoto

**Affiliations:** 1 Department of Rehabilitation, Faculty of Health Sciences, Nihon Fukushi University, Handa, Aichi, Japan; 2 Department of Hygiene, School of Medicine, Fujita Heath University, Toyoake, Aichi, Japan; 3 SDB Research Laboratory, Takaoka Clinic, Nagoya, Aichi, Japan; 4 Division of Respiratory Medicine, Fujita Health University Okazaki Medical Center, Okazaki, Aichi, Japan; 5 Department of Respiratory Medicine, School of Medicine, Fujita Health University, Toyoake, Aichi, Japan; 6 Tokushige Kokyuki Clinic, Nagoya, Aichi, Japan

**Keywords:** Obstructive sleep apnea, Continuous positive airway pressure, Adherence, Diabetes mellitus, Hypertension

## Abstract

**Objectives:**

Continuous positive airway pressure (CPAP) is the first line of therapy for obstructive sleep apnea (OSA). Adherence to CPAP, however, is known to be problematic, and its associations with the comorbidities of hypertension and diabetes mellitus have not been sufficiently evaluated. Thus, we investigated the associations of CPAP therapy adherence with comorbidities of hypertension and diabetes mellitus.

**Methods:**

We conducted a retrospective study among 497 male patients with OSA on CPAP therapy. Participants with pretreatment Apnea–Hypopnea Index (AHI) data based on overnight polysomnographic recordings completed a questionnaire. Adherence data for CPAP therapy were collected using a smart card system. We classified CPAP use of ≥4 hours per night and ≥70% of nights as good adherence; other CPAP use was categorized as poor adherence. Logistic regression models were used to calculate odds ratios (ORs) and 95% confidence intervals (CIs) for poor adherence to CPAP therapy in the hypertension and diabetes mellitus groups, compared with the no comorbidity group, adjusting for body mass index, duration of CPAP therapy, AHI, and Epworth Sleepiness Scale score.

**Results:**

In the no comorbidity, hypertension, and diabetes mellitus groups, 43.4%, 44.7%, and 56.0%, respectively, had poor adherence to CPAP therapy. Being in the diabetes mellitus group was significantly associated with poor adherence to CPAP therapy (OR=1.86, 95% CI: 1.18–2.92, *p*=0.007); there was no association for the hypertension group.

**Conclusion:**

Our results indicate that comorbidity of diabetes mellitus is associated with poor adherence to CPAP therapy in male patients with OSA.

## Introduction

Obstructive sleep apnea (OSA) is a common disorder that is characterized by repetitive upper airway collapse during sleep.^1^ In 2019, 936 million adults aged 30–69 years were estimated to have mild-to-severe OSA (Apnea–Hypopnea Index [AHI] ≥5/hour) and 425 million adults were estimated to have moderate-to-severe OSA (AHI ≥15/hour) globally. Additionally, in the same year, in Japan, the number of people with OSA was approximately 22 million (32.7% of the population aged 30–69 years), and the number of people with severe OSA (AHI ≥15/hour) was approximately 9.4 million (14.0%).^2^ OSA leads to increases in metabolic diseases, hypertension, neurological disorders, cardiovascular diseases, and mortality rates.^3–11^

Continuous positive airway pressure (CPAP) is the first-line therapy for OSA. Maintaining good adherence is essential for fully achieving the therapeutic benefits of CPAP therapy and improving subjective symptoms (such as daytime sleepiness) and high blood pressure.^12–16^

Several studies have reported low adherence to CPAP therapy among patients with OSA and have pointed out several related factors including AHI, Epworth Sleepiness Scale (ESS) score, snoring, and body mass index (BMI).^17–21^ However, adherence to CPAP therapy in patients with OSA and other risk factors for cardiovascular disease, including the comorbidities of hypertension and diabetes mellitus, has not been sufficiently evaluated.^17,22–24^

In the present study, we examined the associations between adherence to CPAP therapy and the comorbidities of hypertension and diabetes mellitus in male patients with OSA, using adherence data recorded using a CPAP device.

## Methods

### Participants

Our participants were Japanese male patients with OSA who were being treated with CPAP at SDB Research Laboratory, Takaoka Clinic, in August 2011. Of the 998 patients who agreed to participate in the study when they visited the clinic, 705 returned a completed questionnaire (see details below) by mail. Adherence data in the participants’ electronic medical records were collected from a smart card, which the participants who returned the questionnaire brought with them when they visited the clinic. The questionnaire collection rate was 71% (705/998), and the adherence data collection rate was 82% (579/705). The mean (standard deviation [SD]) age and AHI of the 705 participants who completed the questionnaire were 56.0 (11.2) years and 49.0 (26.0) events/hour, respectively. The mean (SD) age and AHI of the 579 participants who completed the questionnaire and had valid data on adherence to CPAP therapy were 56.0 (11.1) years and 49.8 (26.0) events/hour, respectively. We excluded 13 patients with missing data on the questionnaire, 35 female patients, and 36 patients with a history of cerebral or cardiovascular disease. Thus, a total of 497 patients with valid data were included in the analysis.

### Survey procedure

Participants were asked to complete a self-administered questionnaire in August 2011. The questionnaire included the ESS;^25^ smoking, drinking, and sleeping habits; and medical history. Weight was also self-reported on the questionnaire. The patients’ AHI, age, and height before CPAP therapy were obtained from clinical records at SDB Research Laboratory, Takaoka Clinic. BMI was calculated as weight divided by the square of height (kg/m^2^).

AHI before CPAP therapy was based on overnight polysomnography recordings (Embla N7000, Embla Systems, Inc., Broomfield, CO, USA), which included continuous electroencephalogram, oculogram, electrocardiogram, electromyogram, respiratory channels including nasal and oronasal airflow, thoracic and abdominal respiratory movements and pulse oximetry, snoring, position, and video monitoring. Sleep stages and respiratory events were scored using the standard diagnostic criteria of the American Academy of Sleep Medicine and a criterion published by registered polysomnogram technicians.^1^ Participants in this study were male patients with OSA who had an AHI ≥20/hour or 5/hour to <20/hour and a failed oral appliance.^26^ Participants were not included if the oral appliance worked.

We used the self-administered questionnaire to ask participants with OSA about their medical history, including their history of the following comorbidities: hypertension, diabetes mellitus, cardiovascular disease, cerebral disease, arrhythmia, and pulmonary hypertension. For each participant, data on adherence to CPAP therapy were obtained for 1 month following the date the questionnaire was completed; these data were objectively measured using a CPAP device with a smart card system. Adherence to CPAP therapy was defined as the proportion of nights CPAP was used and the median number of hours of nightly CPAP use. We classified CPAP use of ≥4 hours per night and ≥70% of nights as good adherence, and other CPAP use was classified as poor adherence.^27^

### Data analyses

Of the 497 participants, 103 had hypertension (hypertension group), 125 had diabetes mellitus (diabetes mellitus group), and 288 had no comorbidities (no comorbidity group). A total of 32 patients with both hypertension and diabetes mellitus were included in the hypertension and diabetes mellitus groups. Average values of age, duration of CPAP therapy, BMI, AHI, and ESS score were compared across the hypertension, diabetes mellitus, and no comorbidity groups using independent samples *t*-tests. Logistic regression models were used to calculate the odds ratios (ORs) and 95% confidence intervals (CIs) for poor adherence to CPAP therapy in the hypertension group and diabetes mellitus group, compared with the no comorbidity group, adjusting for age, duration of CPAP therapy, BMI, AHI, and ESS score.^17–21^ The level of statistical significance was set at 0.05. The analyses were performed using IBM SPSS, Version 23 for Windows (IBM Japan Ltd., Tokyo, Japan).

### Ethical considerations

All participants provided informed consent in writing before completing the self-administered questionnaire. This study was approved in November 2010 by the Ethical Review Board for Epidemiological and Clinical Studies of the Fujita Health University School of Medicine, Aichi, Japan (10-198), and was conducted in accordance with the Declaration of Helsinki.

## Results

The characteristics of the participants in the hypertension, diabetes mellitus, and no comorbidity groups are shown in Table 1. Compared with the values in the no comorbidity group, the average values of age and BMI in the hypertension group and age in the diabetes mellitus group were significantly higher. The average value of BMI was significantly lower in the diabetes mellitus group than in the no comorbidity group (Table 1).

The percentages with poor adherence to CPAP therapy in the no comorbidity, hypertension, and diabetes mellitus groups were 43.4%, 44.7%, and 56.0%, respectively. The ORs and 95% CIs for poor adherence to CPAP therapy in the diabetes mellitus and hypertension groups are shown in Table 2. After adjusting for age, duration of CPAP treatment, BMI, AHI, and ESS score, there was a significant association between being in the diabetes mellitus group and poor adherence to CPAP therapy (OR=1.86, 95% CI: 1.18–2.92, *p*=0.007). There was no association between being in the hypertension group and poor adherence (OR=1.08, 95% CI: 0.67–1.74, *p*=0.751) (Table 2).

## Discussion

Using adherence data recorded using a CPAP device, we found that the comorbidity of diabetes mellitus was significantly associated with poor adherence to CPAP therapy among male patients with OSA and that the comorbidity of hypertension was not significant. There have been several previous studies on diabetes mellitus, hypertension, and adherence to CPAP therapy in patients with OSA.^17,22–24^ One of these studies showed that hypertension was associated with good adherence to CPAP therapy in non-sleepy patients with moderate-to-severe OSA.^23^ Another study reported no association between diabetes mellitus and poor adherence to CPAP therapy in asymptomatic hypertensive patients with OSA.^22^ Another of these studies reported that diabetes mellitus was associated with poor adherence to CPAP therapy in patients with severe OSA.^17^ Additionally, diabetes mellitus was found not to be predictive of CPAP therapy in patients with cardiovascular disease.^24^ Our subjects did not include patients with cardiovascular disease. Our results confirmed the positive association between diabetes mellitus and poor adherence to CPAP therapy in patients with OSA. This finding suggests that, in patients with OSA and diabetes mellitus, health professionals should be aware of poor adherence to CPAP therapy to prevent the development of cardiovascular disease.

There are several possible reasons for the positive association between diabetes mellitus and poor adherence to CPAP therapy in patients. In our study, the patients who had diabetes mellitus may have had poor adherence to diabetes treatment as well as to CPAP therapy, and the patients who had hypertension may have had good adherence to hypertension treatment. Many of the patients with hypertension may have been severely ill and taking antihypertensive drugs; therefore, these patients may have been highly conscious of their treatment. However, the patients who had diabetes mellitus may have had a mild condition, and few were taking medication or insulin injections. Therefore, these patients may have had poor awareness of CPAP therapy. The mean (SD) systolic/diastolic blood pressure in the no comorbidity group, hypertension group, and diabetes mellitus group in this study was 125.3 (11.7)/74.7 (9.6) mmHg, 128.3 (11.4)/74.8 (9.2) mmHg, and 126.8 (12.7)/74.1 (10.5) mmHg, respectively. There was good adherence to hypertension treatment in the hypertension group. There were no data available on HbA_1_c levels or the type of treatment for the hypertension and diabetes mellitus groups. Therefore, these data should be included in future studies. Many previous studies have reported that the proportion of poor adherence to diabetes mellitus treatment among patients with this disease is high and that improvement requires intervention using a multifactorial approach addressing attitudes regarding therapy, self-efficacy, and health education.^28–33^ Improvement in adherence to CPAP therapy among patients with OSA who have diabetes mellitus might require the adoption of more comprehensive approaches.

This study has several limitations. Our study was conducted at a single sleep-disordered breathing clinic using a retrospective design. Our subjects were patients who continued medical treatment for OSA and did not include those who discontinued treatment. We were not able to examine discontinuation status of CPAP treatment. We analyzed 579 of the 998 patients with OSA treated at the clinic during the study period; no information on comorbidities or adherence to CPAP was available for the other patients. The effect of this lack of data on our results is unknown. In our study, the questionnaire collection rate was 71%, and the adherence data collection rate was 82%. Questionnaires were collected by mail, and adherence data were collected from smart cards that participants brought with them when they visited the clinic. The collection rate was not high for either type of data, but the mean age and AHI were similar between the 705 participants who completed the questionnaire and the 579 who had valid data on CPAP therapy adherence. All participants in this study were male patients because there were few female patients at the studied clinic. Although the percentage of patients with OSA who are female is not high, an additional study with data on more female subjects would be important.^2,34^

The adjustment factors were selected on the basis of previous studies; these factors included age, duration of CPAP therapy, BMI, AHI, and ESS score.^17–21^ Including other factors that may have influenced our results (e.g., sleep quality, subjective symptoms, socioeconomic status, level of education, periodic limb movement in sleep index, and residual AHI)^17,34–36^ might have been helpful. However, this information was not available. The participants in this study were receiving ongoing CPAP therapy, and we could not evaluate patients who stopped CPAP therapy. There is no unified definition of adherence, and several studies have reported the prevalence of poor adherence among people being treated with CPAP as 30%–65%.^17–21^ We used a common definition of poor adherence participants using CPAP for <4 hours per night or <70% of nights.^27^

In conclusion, our results indicate that the comorbidity of diabetes mellitus is associated with poor adherence to CPAP therapy in male patients with OSA.

## Figures and Tables

**Table1:** Characteristics of male patients with OSA comorbid with hypertension or diabetes mellitus versus those with no comorbidities

Characteristic (*n*=497)	No comorbidity group (*n*=288)	Hypertension group (*n*=103)	*p* value	Diabetes mellitus group (*n*=125)	*p* value
Age (SD), years	53.4 (10.8)	56.2 (9.8)	0.020	59.1 (10.4)	<0.001
Duration of CPAP therapy (SD), years	2.8 (2.9)	3.1 (3.3)	0.392	3.0 (2.8)	0.714
BMI (SD), kg/m^2^	28.2 (4.9)	30.1 (6.2)	0.001	27.8 (4.5)	0.026
AHI (SD), events/hour	50.8 (23.3)	49.9 (25.4)	0.742	50.8 (20.5)	0.269
ESS score (SD)	8.7 (4.9)	8.8 (4.7)	0.773	9.6 (5.5)	0.164
Habitual sleep (SD), hours	5.9 (1.0)	6.0 (1.3)	0.610	5.9 (1.3)	0.418
Current smoker (%)	60 (20.8)	24 (23.3)	0.601	17 (13.6)	0.083
Non-alcohol drinker (%)	86 (30.0)	28 (27.2)	0.608	27 (21.6)	0.084

Abbreviations: OSA, obstructive sleep apnea; BMI, body mass index; AHI, Apnea–Hypopnea Index; ESS, Epworth Sleepiness Scale; CPAP, continuous airway positive pressure; SD, standard deviation

**Table2:** Associations of adherence to CPAP therapy with diabetes mellitus and hypertension in male patients with OSA

Comorbidity (*n*=497)	Good adherence		Poor adherence				
	*n*		*n*	Percentage (%)	OR	Adjusted OR (95% CI)	*p* value
No comorbidities	163		125	43.4			
Hypertension	57		46	44.7	1.05	1.08 (0.67–1.74)	0.751
Diabetes mellitus	55		70	56.0	1.66	1.86 (1.18–2.92)	0.007

Abbreviations: OSA, obstructive sleep apnea; OR, odds ratio for adherence; CI, confidence interval; CPAP, continuous airway positive pressure
